# Heat Transfer Performance of Functionalized Graphene Nanoplatelet Aqueous Nanofluids

**DOI:** 10.3390/ma9060455

**Published:** 2016-06-08

**Authors:** Roberto Agromayor, David Cabaleiro, Angel A. Pardinas, Javier P. Vallejo, Jose Fernandez-Seara, Luis Lugo

**Affiliations:** 1 Área de Máquinas y Motores Térmicos, Escola de Enxeñería Industrial, Universidade de Vigo, Vigo E-36310, Spain; rober.agro@gmail.com (R.A.); anxo.alvarez.p@gmail.com (A.A.P.); jvallejo@uvigo.es (J.P.V.); jseara@uvigo.es (J.F.-S.); 2 Departamento de Física Aplicada, Facultade de Ciencias, Universidade de Vigo, Vigo E-36310, Spain; dacabaleiro@uvigo.es

**Keywords:** heat transfer coefficient, nanofluid, graphene nanoplatelets, pressure drop

## Abstract

The low thermal conductivity of fluids used in many industrial applications is one of the primary limitations in the development of more efficient heat transfer systems. A promising solution to this problem is the suspension of nanoparticles with high thermal conductivities in a base fluid. These suspensions, known as nanofluids, have great potential for enhancing heat transfer. The heat transfer enhancement of sulfonic acid-functionalized graphene nanoplatelet water-based nanofluids is addressed in this work. A new experimental setup was designed for this purpose. Convection coefficients, pressure drops, and thermophysical properties of various nanofluids at different concentrations were measured for several operational conditions and the results are compared with those of pure water. Enhancements in thermal conductivity and in convection heat transfer coefficient reach 12% (1 wt %) and 32% (0.5 wt %), respectively. New correlations capable of predicting the Nusselt number and the friction factor of this kind of nanofluid as a function of other dimensionless quantities are developed. In addition, thermal performance factors are obtained from the experimental convection coefficient and pressure drop data in order to assess the convenience of replacing the base fluid with designed nanofluids.

## 1. Introduction

Energy is one of the most valuable resources in our society and, therefore, during the last years, many efforts have been made to increase energy efficiency and encourage the use of renewable energies. A wide variety of applications, such as those involved in the field of solar and geothermal energy, entails thermal energy transference from one fluid to another. Thus, heat management has emerged as one of the fields with the highest potential to improve thermal performance. In order to develop more efficient and compact heat transfer equipment, many heat transfer enhancement techniques have arisen since the mid-twentieth century. However, the low thermal conductivity of the fluids commonly used in industrial applications, such as water or glycols, has hindered any heat transfer enhancement. To overcome this problem, several authors have suggested the use of fluids with improved thermal properties.

Based on the fact that solids exhibit thermal conductivities various orders of magnitude larger than conventional heat transfer industrial fluids, in the last decades of the 20th century some researchers proposed enhancing the thermal properties of fluids by dispersing millimetric and micrometric high-conductivity solid particles. Despite the thermal conductivity of these mixtures improving significantly with the addition of solid particles, these dispersions present multiple drawbacks that limit their use in industrial applications. Some of these problems are the erosion of heat transfer equipment, the sedimentation of the solid particles, or the huge increase of pressure drop associated with the higher viscosity in relation to the base fluid. Thus, the addition of solid particles was rejected as a heat enhancement alternative until the last years of the 20th century, when advances in materials science allowed the large-scale production of much smaller solid particles. In 1995, Choi and Eastman [1] resumed previous investigations about the increase of thermal conductivity through the addition of nanometric solid particles to base fluids. These samples, which they named nanofluids, have higher thermal conductivities than the initial base fluids and also lack the problems of the dispersions with millimetric and micrometric particles. Due to the small size of nanoparticles, heat transfer systems are not worn away and the sedimentation is much slower. Moreover, for small concentrations of nanoparticles, pressure drop increases are marginal compared with those of the base fluid [2,3]. The number of experimental studies and publications related to the heat transfer characteristics of nanofluids has increased exponentially in recent years [4,5,6,7]. Among the commonly used base fluids, water is one of the most widespread in heat exchange processes. For this reason, many water-based nanofluid studies have been carried out lately [8,9,10].

Despite the fact that nanofluids are two-phase systems (solid and liquid phases), many authors such as Pak and Cho [11], Xuan and Li [7], and Fard *et al.* [12] agree that their behavior is similar to that of a pure substance with increased thermal properties. In this work, the nanofluids studied are considered as a single-phase system. This approach assumes that the fluid phase and the nanoparticles are in thermal equilibrium and there is no slip in velocity between the base fluid and the solid particles. 

Nanoparticles of different materials such as metals, metallic oxides, and carbon nanostructures have been employed to prepare nanofluids with enhanced thermal properties for many different applications in areas like thermal engineering, renewable energies, or medicine [13,14,15]. The two-dimensional structure of graphene, as a single-atom-thick nanosheet of sp2-bonded carbon atoms packed into a honeycomb lattice, exhibits a higher potential than other nanostructured carbon allotropes, such as one-dimensional nanotubes or zero-dimensional fullerenes [16]. Thus, graphene combines the advantages of highly ordered graphitic carbonhigh stability, abundance in source, and low cost—with the single-layer benefits of in-plane thermal conductivity as high as 3000 to 5000 W·m^−1^·K^−1^ [16,17,18]. Although graphene is hydrophobic and, consequently, cannot be dispersed in water for a long time without agglomerating [19], stable dispersions in aqueous/organic solvents can be prepared once the material has been functionalized (using an acid treatment or amino function) by means of proper sonication [20,21,22]. Yarmand *et al.* [22] stated that water nanofluids based on functionalized graphene nanoplatelets and silver (up to 0.1 wt %) are stable without the assistance of surfactants or ultrasonication.

Most of the studies about nanofluids focus on the analysis of their physical properties, both from an experimental and a theoretical point of view. The properties that play a major role in convection heat transfer are density, specific heat capacity, thermal conductivity, and viscosity. Although density and specific heat capacity can be easily predicted from a theoretical approach, thermal conductivity and viscosity must be carefully determined because there are not yet models that can accurately predict the transport properties of nanofluids [23,24]. Over the last years, several studies about the thermophysical and heat transfer performance of graphene nanoplatelet aqueous nanofluids have been reported in the literature [22,25,26,27,28,29,30]. However, most of them only analyze the effect of nanoplatelet addition at low mass concentrations. Thus, regarding thermophysical characterization, only Gupta *et al.* [25] and Hajjar *et al.* [26] reached 0.25 wt %, while Yu *et al.* [27] studied from 1 to 5 vol %.

Forced convection heat transfer is a complex phenomenon that combines velocity and temperature profiles, as well as the thermal properties of the fluid. Significant heat transfer enhancements are expected with the addition of high-conductivity nanometric particles. The increase of the effective thermal conductivity of the fluid is not the only factor that accelerates the heat transfer process [31]. The presence of these solid particles also increases the fluctuation and turbulence of nanofluids, further enhancing the heat transfer process. The increase of turbulence causes temperature gradients within the fluid to be reduced and the boundary layers to become thinner where transport phenomena are slower. Similarly, thanks to the small size of the solid particles, the increase of the pressure drop with respect to the base fluid is minimal. These characteristics of the fluid flow and heat transfer of nanofluids were also discussed by Xuan and Li [9]. Consequently, greater energy exchanges between the fluid and the solid walls of heat transfer equipment can be achieved for the same pumping power input. This is the reason why the use of nanofluids is one alternative with high potential for improving the energy efficiency of heat transfer processes. One of the current trends consists of the study of the effects and potential benefits resulting from the application of magnetic fields in heat transfer processes with nanofluids [10,32,33,34,35,36].

A difficulty faced when studying nanofluids is that the conventional heat transfer coefficient correlations of pure substances fail to predict their thermal behavior, even if the measured transport properties of the nanofluids are used in the calculations. This problem was previously reported by Xuan and Li [9], among others. It is caused by the presence of moving solid particles that modify the velocity and temperature profiles of the flow with respect to those of pure substances. Thus, new correlations that account for the presence of solid particles must be explored in order to quantitatively describe the thermal behavior of nanofluids.

The aim of this paper is to study the single-phase flow and thermal behavior of different sulfonic acid-functionalized graphene nanoplatelet aqueous nanofluids from an experimental point of view. Nanoplatelets and nanofluids are thermophysically characterized, and the heat transfer by forced convection is studied for different nanoparticle concentrations using a new device. The main results are discussed and compared with those obtained for water at the same operating conditions to investigate the heat transfer enhancement. Finally, new correlations to calculate the Nusselt number and the Darcy friction factor of this kind of nanofluids are proposed.

## 2. Nanofluid Characterization

### 2.1. Material and Sample Preparation

Graphene nanoplatelets (GnPs) were provided by NanoInnova Technologies S.L. (Madrid, Spain) while Milli Q-Grade water was produced with a resistivity of 18.2 MΩ·cm by means of a Millipore system Milli-Q 185 Plus (Millipore Ltd., Watford, UK). Nanofluids were designed through a two-step method as dispersions of sulfonic acid-functionalized graphene nanoplatelets at nanoadditive mass concentrations of 0.25, 0.50, 0.75, and 1.00 wt % in water, which correspond to volume fractions of 0.19, 0.39, 0.59, and 0.79 vol %, respectively. The desired concentrations were prepared by weighing the powder in a Mettler AE-200 (Mettler Toledo, Greifensee, Switzerland), with an uncertainty of 10^–4^ g, and then stirring it into a predetermined volume of base fluid for 120 min. Afterwards, the suspensions were sonicated for 240 min by using an ultrasonic bath (Ultrasounds, JP Selecta S.A., Barcelona, Spain) operating at a sonication frequency of 20 kHz, with a maximum power output of 200 W.

### 2.2. Nanopowder Characterization

**Figure 1:**
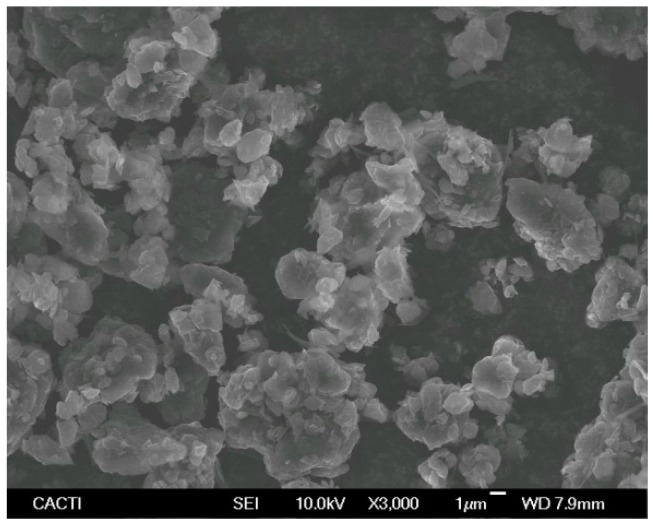
SEM image of sulfonic acid-functionalized graphene nanoplatelets at 3000× magnification.

**Figure 2:**
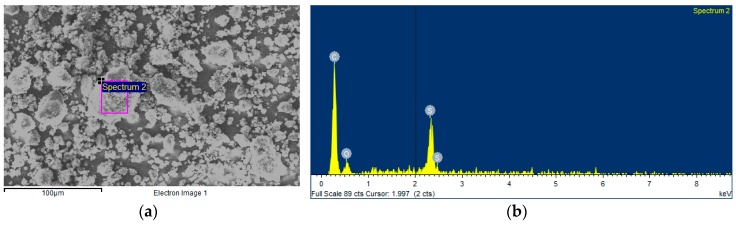
EDS microanalysis of sulfonic acid-functionalized graphene nanoplatelets: (**a**) studied area; (**b**) EDS spectrum.

The morphology of the dry sulfonic acid-functionalized graphene nanopowder was analyzed through scanning electron microscopy by using a JEOL JSM-6700F field emission gun-SEM (JEOL, Tokyo, Japan) working at an accelerator voltage of 20 kV in backscattering electron image (Yttrium Aluminum Garnet type detector). This device is coupled to an energy dispersive X-ray (EDS) spectrometer Oxford Inca Energy 300 SEM (Oxford Instruments, Oxford, UK), which also allows for carrying out chemical microanalyses of the sample. SEM samples were prepared by depositing a drop of the nanopowder dispersed in analytical grade methanol (Sigma-Aldrich, Madrid, Spain) on the top of a silica support and drying under atmospheric conditions. Figure 1 and Figure 2 show the SEM image and EDS microanalysis of the studied GnP sample, respectively. Sulfonic acid-functionalized graphene exhibits a nanoplatelet-shape of up to some micrometers with wrinkled surfaces folding at the edges (Figure 1). The EDS spectrum shows the presence of carbon (C), oxygen (O), sulfur (S), and silica (Si), this last being due to impurities of physical support (Figure 2).

### 2.3. Thermophysical Characterization

**Figure 3:**
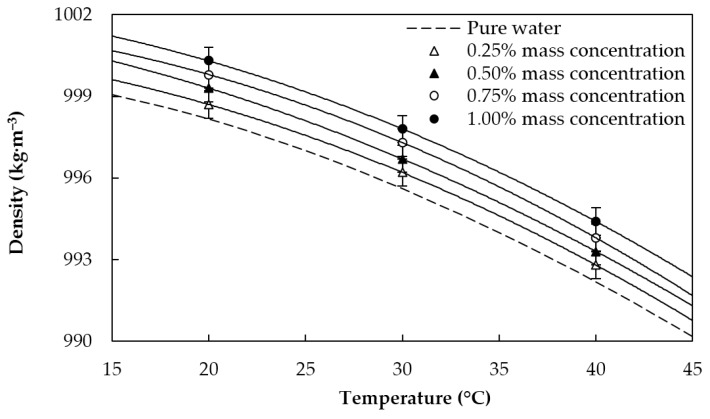
Density of the sulfonic acid-functionalized graphene nanoplatelet water-based nanofluids and water [37].

**Figure 4:**
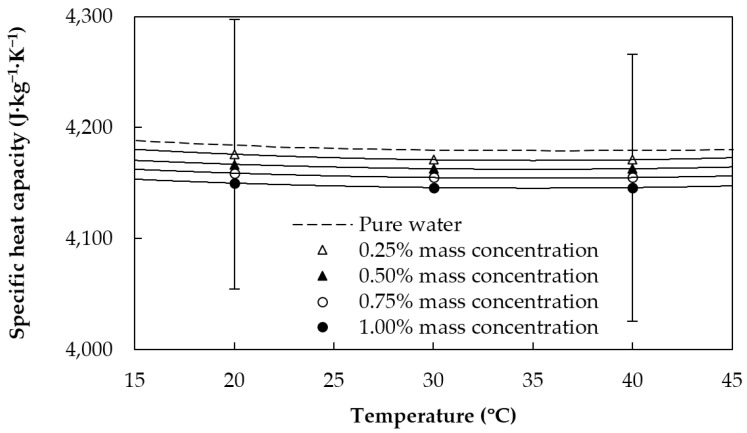
Specific heat capacity of the sulfonic acid-functionalized graphene nanoplatelet water-based nanofluids and water [37].

Regarding the thermophysical properties of the base fluid, those values from REFPROP [37] were considered. Concerning the dry GnP sample, the specific heat capacity was experimentally determined in this work by a quasi-isothermal temperature-modulated differential scanning calorimetry method (TDMSC) using a heat-flux differential scanning calorimeter, DSC, Q2000 (TA Instruments, New Castel, DE, USA) [38]. Otherwise, a density value of 1.27 g·cm^−3^ for GnP nanopowder was used. In this work, the densities, *ρ*, and specific heat capacities, *c_p_*, of GnP nanofluids were obtained using the following weighted average equations [38]:

(1) $$\rho_{nf}=\phi_{v}\times \rho_{GnP}+\left(1-\phi_{v}\right)\times \rho_{bf}$$

(2) $$c_{p\mathrm{nf}}=\phi_{m}\times c_{p\mathrm{GnP}}+\left(1-\phi_{m}\right)\times c_{p\mathrm{bf}}$$

where *φ_v_* is the nanoadditive volume fraction, *φ_m_* is the nanoadditive mass fraction, and the *nf*, GnP, and bf subscripts stand for nanofluid, graphene nanoplatelets, and base fluid, respectively. Obtained values for the density and specific heat capacity of the studied fluids in the temperature range from 20 to 40 °C are plotted together with the base fluid in Figure 3 and Figure 4, respectively. Density increases with increasing graphene concentration, whereas the contrary occurs for the specific heat capacity. Modifications in the mentioned properties as regards the base fluid overall analyzed conditions are lower than 0.2% for *ρ* and 0.8% for *c_p_*.

**Figure 5:**
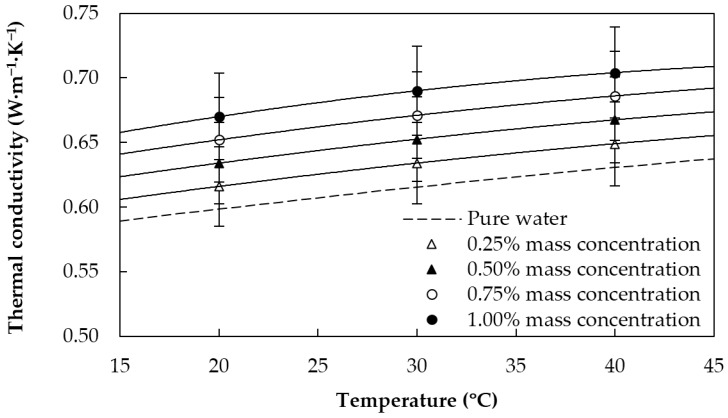
Thermal conductivity of the sulfonic acid-functionalized graphene nanoplatelet water-based nanofluids and water [37].

Effective thermal conductivities, k, were experimentally measured in this work from 20 to 40 °C by means of a KD2 Pro thermal analyzer (Decagon Devices, Inc., Pullman, WA, USA) together with a KS-1 probe of 1.3 mm diameter and 60 mm long. In order to precisely control the temperature, samples were fully immersed in a Grant GP200 (Grant Instruments, Cambridge, UK) oil bath. More details about the experimental device and measurement procedure can be found in previous works [39,40,41]. The temperature dependence of the thermal conductivity of the base fluid and the different GnP nanofluids is shown in Figure 5. This property rises as the nanoplatelet concentration increases, being 12% greater for the 1.00 wt % nanofluid than for the base fluid.

**Figure 6:**
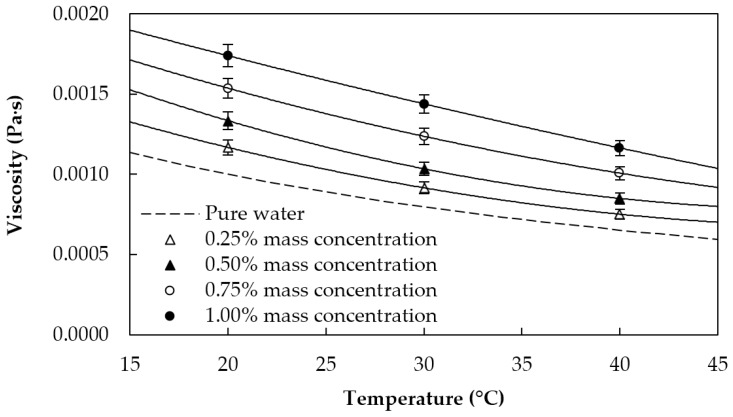
Viscosity of the sulfonic acid-functionalized graphene nanoplatelet water-based nanofluids and water [37].

Finally, the dynamic viscosities of the fluid samples were determined in the temperature range between 20 and 40 °C by using a rotational Physica MCR 101 rheometer (Anton Paar, Graz, Austria) equipped with a cone-plate geometry with a cone angle of 1° and a diameter of 25 mm [2,41]. Both nanofluids and base fluid exhibit Newtonian behavior in the studied concentration range. Obtained values are presented in Figure 6 as a function of the temperature, where a remarkable increase in viscosity with the mass concentration can be seen.

## 3. Heat Transfer Coefficient Determination

### 3.1. Experimental Equipment

**Figure 7:**
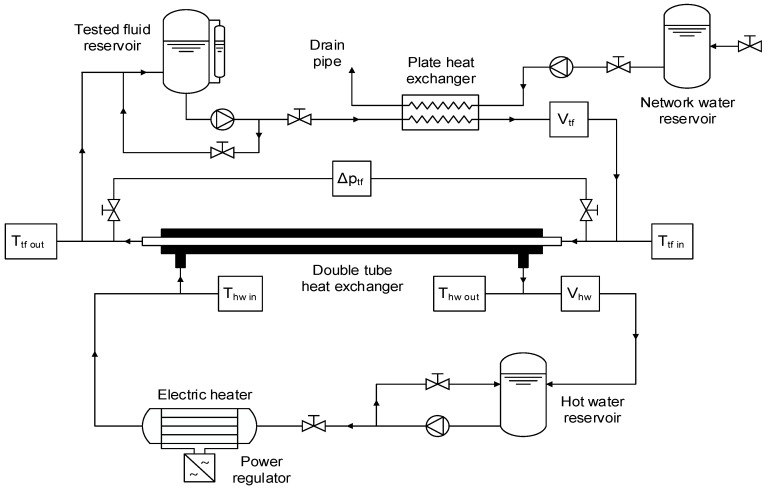
Layout of the experimental setup for the determination of heat transfer coefficients and pressure drop.

The new experimental facility is composed of two closed loops, using the tested fluid and hot water as working fluids, and an open refrigeration loop. The layout of the experimental equipment and the location of the devices used are shown in Figure 7. The main section of the experimental facility, where heat exchange and pressure drop are studied, consists of a stainless steel tube-in-tube heat exchanger with lengths of 930 mm and 1180 mm for the test sections of effective heating and pressure drop, respectively. The tested fluid is pumped through the inner tube and is heated by hot water from the other closed loop, which flows within the annular section. The internal tube has an inner diameter of 8 mm and an outer diameter of 10 mm, while the outer tube has an inner diameter of 15 mm. The test section was insulated to minimize the heat losses to the environment, which were estimated to be lower than 0.5% of the total heat transfer even for the most unfavorable operating conditions. Thus, the heat exchanger can be considered adiabatic, which greatly simplifies the analysis.

The hot water is stored in a 25 L reservoir that gives thermal inertia to the loop and is pumped through a 4.5 kW electric heater before it enters the annular section. Similarly, the tested fluid is pumped from a 3 L reservoir tank to a plate heat exchanger, where it transfers energy to the water of refrigeration. This tested fluid then flows into the tube-in-tube heat exchanger, where it receives energy from the hot water. The refrigeration loop consists of a reservoir tank where tap water is stored before being pumped through the plate heat exchanger to cool the tested fluid.

The flow rates of the hot water and tested fluid are adjusted both with valves and controlling the rotation speed of the centrifugal pumps with proportional-integral-derivative (PID) regulators. The temperature of the hot water is also controlled automatically with a PID that adjusts the electric power delivered by a power regulator to the electric heater. The flow rate of refrigeration water, and therefore the temperature of the tested fluid, is controlled manually with a needle valve.

The temperatures of hot water and tested fluid at the inlet and outlet of the test section are measured with four Pt100 A Class resistance temperature sensors. The flow rate of both fluids is measured with two different electromagnetic flowmeters. The pressure drop of the tested fluid through the tube-in-tube heat exchanger is measured with a differential pressure sensor. Lastly, the experimental setup is equipped with a data acquisition system based on a 16-bit acquisition card and a PC where the measured variables are displayed in real time and stored in a spreadsheet.

### 3.2. Experimental Procedure

With the aim of evaluating the enhancement of the convection heat transfer coefficients and the pressure drop of several sulfonic acid-functionalized graphene nanoplatelet aqueous nanofluids, a comparison between these results and those obtained for pure water was experimentally carried out. Thus, several sets of tests were conducted for nanofluids at 0.25%, 0.50%, 0.75%, and 1.00% mass concentrations of graphene nanoplatelets, together with those for pure water. Each test is defined through the values of the volumetric flow rate and average temperature in the heat exchanger of each fluid.

Heat transfer tests were conducted by pumping the fluid under study at different flow rates while the average temperature of both hot water and the tested fluid as well as the flow rate of hot water were kept constant. The pairs of studied average temperatures in relation to tested fluid/hot water were, in Celsius, 20/40, 30/40, 30/45, 30/50, and 40/60, and the studied fluid flow rate ranged from 200 to 700 L/h in 100 L/h steps. The hot water flow rate was set to 800 L/h for all the tests. The water flow rate was selected to be as small as possible to achieve a high temperature difference across the heat exchange, but high enough to ensure turbulent regime (Re > 10^4^). This was done in order to calculate the heat transfer coefficient on the water side using widely accepted correlations for forced convection through annular sections. In order to assess the validity of the experimental measurements, the convection heat transfer coefficients for pure water were compared with the values predicted by the well-known Gnielinski correlations [42]. Deviations were less than 10% for all the tested conditions.

The pressure drop tests were also carried out keeping the temperature of the tested fluid constant and pumping at different flow rates. Two tests were conducted for average temperatures of 25 °C and 35 °C and the flow rate was varied from 250 to 700 L·h^−1^ in 50 L·h^−1^ steps. The deviations of the pressure drops measured and those calculated from Gnielinski correlations [42] were also less than 10%, finding slightly higher deviations for the lowest flow rates.

## 4. Data Analysis

The heat transfer capability of the graphene water-based nanofluids can be quantitatively described through the convection heat transfer coefficient. The convection coefficients of the different nanofluids are compared with those of pure water to analyze the heat transfer enhancement. Similarly, the pressure drops of the nanofluids and pure water are also compared.

The convection coefficient cannot be measured directly, but can be calculated from the measured data as follows. The temperatures of the tested fluid and the heating water at the inlet and the outlet of the test section (*T_tf in_*, *T_tf out_*, *T_hw in_*, and *T_hw out_*) were measured experimentally. The logarithmic mean temperature difference (*LMTD*), normally used in heat exchangers to describe the temperature driving force, is defined as:

(3) $$LMTD=\frac{\left(T_{\mathrm{hw}\mathrm{in}}-T_{\mathrm{tf}\mathrm{out}})-(T_{\mathrm{hw}\mathrm{out}}-T_{\mathrm{tf}\mathrm{in}}\right)}{\ln\left(\frac{T_{\mathrm{hw}\mathrm{in}}-T_{\mathrm{tf}\mathrm{out}}}{T_{\mathrm{hw}\mathrm{out}}-T_{\mathrm{tf}\mathrm{in}}}\right)}$$

The overall thermal resistance in the test section, *R_ov_*, was obtained as the quotient of the (LMTD) and the heat flow rate on the hot water side $\left(\dot{Q}\right)$ according to the following equation:

(4) $$R_{ov}=\frac{LMTD}{\dot{Q}}$$

where $\dot{Q}$ is given by:

(5) $$\dot{Q}=\rho_{hw}\times c_{p\mathrm{hw}}\times {\dot{V}}_{hw}\times (T_{\mathrm{hw}\mathrm{in}}-T_{\mathrm{hw}\mathrm{out}})$$

where $\dot{V}$_hw_ is the hot water volume flow rate while *ρ*_hw_ and *c_p hw_* stand for its density and specific heat capacity, respectively. The calculations were carried out using the heat flow in the hot water side, Equation (5), because the uncertainties of the water properties are lower than those of the nanofluids.

The overall thermal resistance is the sum of the thermal resistance corresponding to the external forced convection (*R_hw_*), the conductive thermal resistance of the steel tube (*R_s_*), and the internal forced convection resistance (*R_tf_*). Fouling resistances were assumed to be negligible in this work because the tube used was cleaned before the experiments. Therefore, the thermal resistance on the tested fluid side can be calculated using the following equation:

(6) $$R_{tf}=R_{ov}-R_{s}-R_{hw}$$

The convection thermal resistance of the hot water side is given by the equation:

(7) $$R_{hw}={\left(\pi \times d_{2}\times L_{h}\times h_{hw}\right)}^{-1}$$

where *d*_2_ corresponds to the outer diameter of the inner tube and *L_h_* is the effective heating length of the test section. The heat transfer coefficient, *h_hw_*, was calculated using the correlations proposed by Gnielinski [42] for fully developed turbulent flows in annular ducts with adiabatic outer surfaces. These relations are given by:

(8) $$h_{hw}=k_{hw}\times Nu_{hw}/d_{h}$$

(9) $$Nu_{hw}=\psi \times Nu^{*}{}_{hw}$$

(10) $$\psi =0.75\times a^{-0.17}$$

(11) $$a=d_{2}/d_{3}$$

(12) Nu∗w=(fhw/8)×Rehw×Prhw×(1+(dh/Lh)2/3)ω+12.7×(fhw/8)1/2×(Prhw2/3−1)

(13) ω=1.07+900×Rehw−1−0.63×(1+10×Prhw)−1

(14) fhw=(0.782×ln(Re∗hw)−1.50)−2

(15) Re∗hw=(1+a2)×ln(a)+(1−a2)(1−a)2×ln(a)×Rehw

where *d*_h_, the hydraulic diameter for the annular section, is the inner diameter of the outer tube, *d*_3_, minus the outer diameter of the inner tube, *d*_2_; and *k_hw_* is the thermal conductivity of hot water. The Nusselt number (*Nu_hw_*), the Reynolds number (Re_hw_), and the Prandtl number (Pr_hw_) of the hot water were calculated according to their usual definitions. The thermophysical and transport properties of hot water were obtained from REFPROP [37], considering water to be a saturated liquid at an average temperature between the inlet and the outlet.

On the other hand, the conductive thermal resistance, *R_s_*, was calculated assuming unidimensional heat transfer in the radial direction and according to the equation:

(16) $$R_{s}=\frac{\ln(d_{2}/d_{1})}{2\pi \times k_{s}\times L_{h}}$$

where *d*_1_ is the inner diameter of the inner tube of the heat exchanger, made of AISI 316L stainless steel. Its thermal conductivity (*k_s_*) was calculated using the equation proposed by Choong [43]:

(17) $$k_{s}=9.248+0.01571\times (T+273.15)$$

where the temperature of the tube, *T*, should be expressed in Celsius and the conductivity in W·m^−1^·K^−1^. The temperature of the tube for each test was considered as a constant given by:

(18) $$T=\frac{1}{4}\times (T_{tf_{}\;in}+T_{tf_{}\;out}+T_{hw_{}\;in}+T_{hw_{}\;out})$$

Consequently, once the inner thermal resistance, *R_tf_*, due to convection was calculated using Equation (6), the convection coefficient was determined in a straightforward way using the following equation:

(19) $$h_{tf}=\pi \times d_{1}\times L_{h}\times R_{tf}{}^{-1}$$

On the other hand, the pressure drop of the fluid under study was measured directly and no further analysis is required to compare the results of the different nanofluids and water.

Heat transfer coefficient and pressure drop are dimensional variables and, although they can be convenient for comparing the thermal and hydrodynamic behavior of the nanofluids, they are not suitable to correlate the experimental data. Instead, dimensionless groups can be used to correlate the heat transfer and pressure drop with the parameters of the flow and the properties and composition of the nanofluid.

The tested fluid Nusselt number (*Nu_tf_*) is the dimensionless form for the convection coefficient of the tested fluid (*h_tf_*) and is given by the following equation:

(20) $$Nu_{tf}=\frac{h_{tf}\times d_{1}}{k_{tf}}$$

Similarly, the Darcy friction factor of the tested fluid (*f_tf_*), which represents the dimensionless pressure drop across the heat exchanger, is given by:

(21) $$f_{tf}=\frac{d_{1}}{L_{p}}\times \frac{\Delta p_{tf}}{\rho_{tf}\times v_{tf}{}^{2}/2}$$

where *L_p_* stands for the effective pressure drop length of the test section while *Δp_tf_*, *ρ_tf_*, and *v_tf_* are the pressure drop, density, and average velocity of the tested fluid, respectively.

In this work, the Nusselt number of the tested fluid (*Nu_tf_*) was related to its Reynolds number (Re*_tf_*), Prandtl number evaluated at the bulk temperature (Pr*_tf_*), Prandtl number evaluated at the wall temperature (Pr*_wall_*), and volumetric concentration of nanoparticles (*φ_v_*). The following equation is proposed in this work to correlate the heat transfer experimental data for the tested fluids:

(22) Nutf=c1×(1+c2×ϕv)c3×Retfc4×Prtfc5×(Prtf/Prwall)c6

The Prandtl number, Pr*_wall_*, at the wall is included in the correlation to account for the heat transfer effect on the temperature profile and so on the thermal properties. The temperature at the wall was considered as a constant given by:

(23) $$T_{wall}=\frac{1}{2}\times (T_{\mathrm{tf}\mathrm{in}}+T_{\mathrm{tf}\mathrm{out}})+R_{tf}\times \dot{Q}$$

It should be noted that the volumetric concentration of nanoparticles was used to correlate the experimental data. This is because the rheological behavior of suspensions, such as nanofluids, highly depends on hydrodynamic forces, which act on the surfaces of the particles; therefore, a geometric way to describe the concentration of the fluid is preferred. This is a common practice when studying nanofluids, as is also discussed by Pak and Cho [11].

Likewise, the Darcy friction factor of the tested fluid (*f_tf_*) was related to the Reynolds number and the volumetric concentration of nanoadditives. The functional relation proposed for the friction factor follows the expression:

(24) ftf=c7×(1+c8×ϕv)c9×Retfc10

In order to further evaluate the thermal performance, the comparison of the Nusselt numbers of two fluids must be balanced by the friction factors, which are influenced by the nanoparticles’ dispersion. A possible means of evaluating the thermal effectiveness of the different tested nanofluids is to use the thermal performance factor given by the following relation [44,45]:

(25) $$\eta_{tf}=(Nu_{tf}/Nu_{bf})\times (f_{tf}/f_{bf}{)}^{1/3}$$

where *f* is the Darcy friction factor, Nu the Nusselt number, and *tf* and *bf* stand for tested and base fluids, respectively. The experimental results were analyzed using this parameter to compare the thermal performance of the different nanofluids.

The required thermophysical properties of the tested fluid, evaluated at the average temperature in the heat exchanger, were obtained from the aforementioned experimental values. A thorough uncertainty analysis of the measured and calculated magnitudes was performed following the recommendations of the “Guide to the Expression of Uncertainty in Measurements (GUM)” [46]. The results of this analysis are summarized later.

## 5. Results

Firstly, the heat transfer coefficients and the pressure losses of the different nanofluids are compared and discussed in relation to water results. After that, the obtained numerical values for the Nusselt number and Darcy friction factor correlations are presented together with their validity ranges. In addition, the analysis of the thermal performance factor as a function of mass concentration is discussed.

### 5.1. Heat Transfer Performance

The experimental results for the analyzed sulfonic acid-functionalized graphene nanoplatelet water-based nanofluids are shown as an example of some of the numerous tests. The displayed behaviors are representative of the rest of the tests performed, the tendency of the data being similar along all the other tests.

#### 5.1.1. Heat Transfer Coefficients

**Figure 8:**
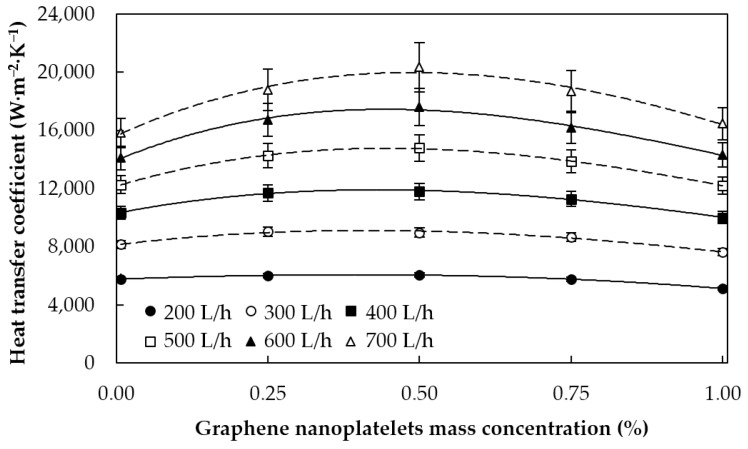
Heat transfer coefficient against graphene nanoplatelet concentration at different flow rates. The average temperatures of tested fluid/hot water are 20 °C/40 °C.

**Figure 9:**
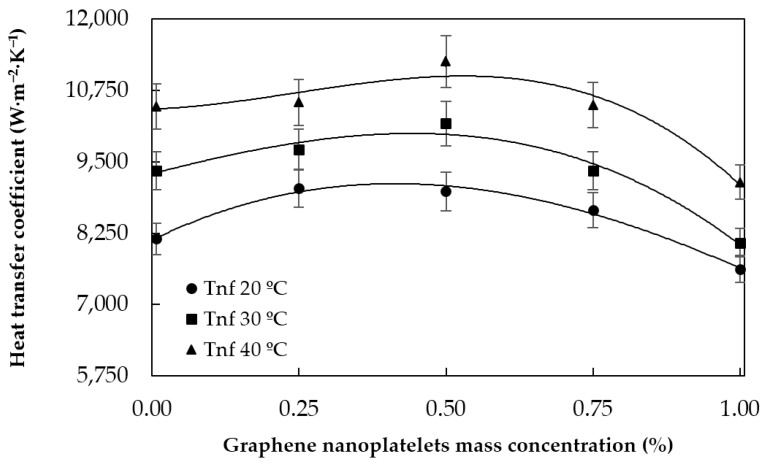
Heat transfer coefficient against graphene nanoplatelet concentration at different temperatures with a flow rate of 300 L·h^−1^. The LMTD at the heat exchanger is 20 °C.

The experimental heat transfer coefficients of the different analyzed nanofluids and pure water in the 20 °C/40 °C tests at different flow rates are plotted in Figure 8 as a function of the mass concentration of graphene nanoplatelets at different flow rates. As could be expected, the heat transfer coefficients increase with the flow rate due to the increase of mixing and turbulence. The heat transfer coefficients for the 300 L·h^−1^ tests at different temperatures are plotted in Figure 9. Each curve represents the values measured at different tested fluid average temperatures, with 20 °C as heat exchanger LMTD. As can be observed, the heat transfer coefficient is higher as the temperature of the nanofluid rises, which agrees with the decrease of the tested fluid viscosity and the increase of its thermal conductivity.

Upon further inspection of Figure 8 and Figure 9, it can be seen that the heat transfer coefficients have a smooth dependence with the concentration of graphene nanoplatelets. The heat transfer achieved with the 0.25% nanofluid is greater than that of pure water and the results were even better when the concentration was 0.50%. However, the heat transfer enhancement obtained with the 0.75% nanofluid was smaller and the heat transfer coefficients for the 1.00% mass concentration were even worse than those of pure water under many conditions. This may be because, at low nanoparticle concentrations, the heat transfer enhancement due to the thermal conductivity and the increased degree of mixing are more important than the penalty caused by the increase of the effective viscosity of the nanofluid. Nevertheless, at higher concentrations, the increase of viscosity due to the presence of solid particles predominates over the other two effects and, as a result, the heat transfer is reduced with respect to that achieved with the base fluid. This suggests the existence of an optimum nanoparticle concentration for a given set of operating conditions. The obtained relative heat transfer enhancements with respect to water for each nanofluid (minimum, average, and maximum) are summarized in Table 1.

In relation to the heat transfer enhancements, we notice that Sadeghinezhad *et al.* [28] obtained increments in the Nusselt number up to 83% for graphene nanoplatelets nanofluids at 0.1 wt % under turbulent forced convection through a plain stainless steel tube. Yarmand *et al.* [29] quantified their maximum enhancement of the Nusselt number at 26.5% using a square stainless steel tube for samples at 0.1 wt %.

#### 5.1.2. Pressure Losses

**Figure 10:**
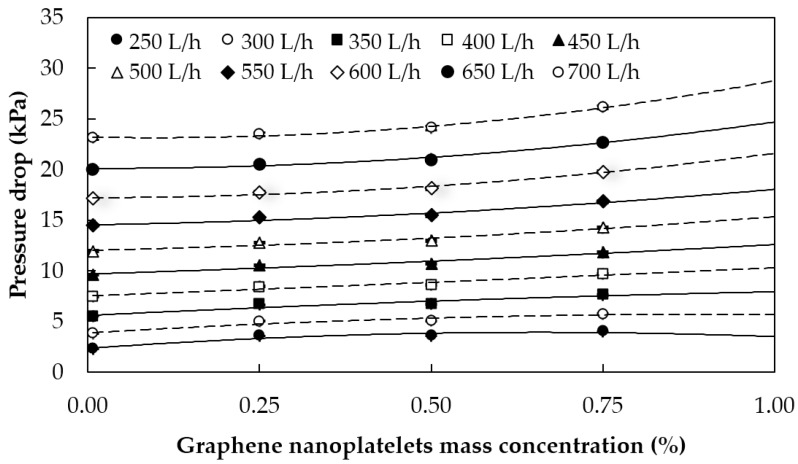
Pressure drops *versus* graphene nanoplatelet concentration at 35 °C test fluid temperature and for different flow rates.

To determine if this kind of nanofluid is suitable for heat transfer applications, the pressure drop caused by the presence of the solid particles should also be analyzed. The measurements of the pressure drop of the 0.25%, 0.50%, and 0.75% nanofluids, as well as those of pure water, for different flow rates and at a test fluid temperature of 35 °C, are plotted in Figure 10, as an example. As expected, the pressure losses increase with the flow rate. Moreover, as can be seen in this figure, the pressure drop slightly increases with the mass concentration. This increase can be due to the higher viscosities of the nanofluid and, as the concentration of graphene increases, this effect becomes more important and the pressure losses rise.

Taking into account all the tests performed, it was also found that at a lower nanofluid temperature, e.g., 25 °C, the pressure losses were slightly higher, as a consequence of the higher viscosities. The relative experimental pressure increases with respect to pure water for each nanofluid (minimum, average, and maximum) are summarized in Table 2.

These results are in agreement with those of Sadeghinezhad *et al.* [28], who found maximum increases in pressure drops of 14.6% for 0.1 wt %.

### 5.2. Dimensionless Analysis

Many correlations to calculate the Nusselt number and the Darcy friction factor for internal forced convection in circular ducts are available in the literature. However, those correlations were developed using measurements of pure substances and are not capable of predicting the behavior of nanofluids. Two new correlations suitable for calculating the Nusselt number and Darcy friction factor of sulfonic acid-functionalized graphene nanofluids were developed in this work.

#### 5.2.1. Nusselt Number Correlation

The *c*_1_–*c*_6_ fitting coefficients in Equation (22) were determined using a least squares regression analysis. The proposed correlation for the Nusselt number and its validity ranges are given by the following equations:

(26) Nunf=0.011×(1+100×ϕv)−0.095×Renf0.886×Prnf0.545×(Prnf/Prwall)−0.495

(27) $$0.19\%\leq \phi_{v}\leq 0.79\%$$

(28) 5×103≤Renf≤4×104

(29) $$4.8\leq {\mathrm{Pr}}_{nf}\leq 10.8$$

(30) $$1.06\leq {\mathrm{Pr}}_{nf}/{\mathrm{Pr}}_{wall}\leq 1.36$$

It should be noted that the factor Pr*_nf_*/Pr*_wall_* that is used to account for the effect that the heat transfer has on the temperature profile is different if the fluid is either heated or cooled. During all the tests the heat was transferred from the hot water to the nanofluid and, therefore, the correlation given by Equation (26) should only be used when the nanofluid is being heated. The uncertainty of the experimental Nusselt number was evaluated and it ranged from 6% to 12% for all the measured values. The deviation between the Nusselt numbers correlated and those measured experimentally is 13.2% for the worst case (0.25% mass concentration nanofluid, 40 °C/60 °C, and 700 L·h^−1^) and is less than 10% and 8% for 90% and 75% of the measured values, respectively.

#### 5.2.2. Darcy Friction Factor Correlation

The procedure for the friction factor correlation was similar to that of the Nusselt number. The obtained fitting parameters from *c*_7_ to *c*_10_ in Equation (24) are:

(31) fnf=0.109×(1+100×ϕv)0.215×Renf−0.159

(32) $$0.19\%\leq \phi_{v}\leq 0.59\%$$

(33) 8×103≤Renf≤3.7×104

The uncertainty analysis of the experimental friction factor leads to values below 2% for all the measured values. The deviation between the Darcy friction factors correlated and those measured experimentally is 4.7% for the worst case (0.75% mass concentration nanofluid, 35 °C, and 300 L·h^−1^) and is less than 2.5% and 2% for 90% and 75% of the measured values, respectively.

#### 5.2.3. Thermal Performance Factor

**Figure 11:**
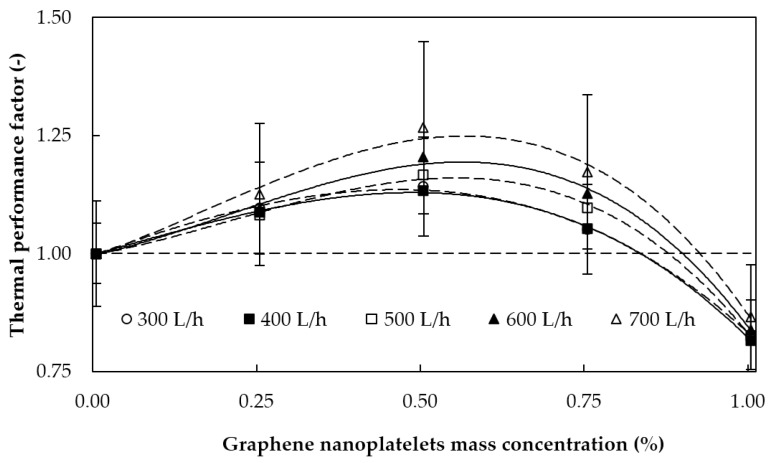
Thermal performance factor against graphene nanoplatelet concentration at 30 °C, with LMTD of 10 °C between fluids, for different flow rates.

An analysis of the thermal performance factor obtained for each graphene nanoplatelet mass concentration was carried out by combining the results of the different tests as gathered in Figure 11 for different flow rates.

A reference temperature of 30 °C for both experiments was selected in order to develop this comparison. Thus, Nusselt numbers were obtained from the 30 °C/40 °C heat transfer test (average temperature of the tested fluid at 30 °C) while friction factors were obtained through an interpolation of both 25 °C and 35 °C pressure drop tests. As can be observed in Figure 11, the thermal performance factor rises when the flow rate increases and all nanofluids present beneficial ratios except for the 1 wt %. The concentration dependence of this factor exhibits maximum values at 0.5% mass concentration, reaching a value of 1.27 for the highest flow rate. In agreement with previous results, the 1% sample shows the worst results, with thermal performance factors below the unit for all the analyzed flow rates.

## 6. Conclusions

In this work, a new experimental device was implemented to determine the heat transfer coefficients and the pressure drops of several sulfonic acid-functionalized graphene nanofluids in water up to 1 wt % (0.79 vol %). A rheometer, a transient hot wire technique, and a differential scanning calorimeter were used to measure viscosities, thermal conductivities, and heat capacities of the analyzed samples, respectively. Enhancements in thermal conductivity reach up to 12% for the maximum analyzed concentration.

Noticeable enhancements in the convection coefficients, *h_tf_*, are achieved for all nanofluids except for 1 wt %. These coefficients increase with the flow rate and the temperature of the fluid. The mass concentration dependence shows an optimum at 0.5%, for which *h_tf_* are up to 32% higher than those for water. The pressure losses monotonously increase with the nanoparticle concentration as a consequence of the viscosity of the tested fluid.

Two new correlations able to describe the Nusselt number and the friction factor of this kind of dispersion as a function of dimensionless numbers are presented. Deviations lower than 13.2% in the case of Nusselt and 4.7% for pressure losses are obtained. Moreover, heat transfer coefficient and pressure drop results were combined through the thermal performance factor to assess whether replacing the base fluid with the different designed nanofluids would be beneficial. The 0.5% nanofluid was found to exhibit the best results, reaching increases up to 27% in this factor. Hence, the use of nanofluids is an alternative with a high potential for heat transfer enhancement considering that, for an appropriate graphene nanoplatelet concentration, significant improvements in the heat transfer coefficients can be achieved, with low pressure drop increases.

The use of nanofluids would allow for developing more efficient and compacting heat transfer equipment. It can be especially significant in renewable and clean energy technologies, with a potentially interesting application in the field of solar and geothermal energy. This type of new material can lead to reductions in the temperature difference between fluids, increasing the efficiency of thermal machinery or decreasing the required flow rates, which would reduce the pumping power consumption. However, the field of nanofluids is still in its early stages and new works, both theoretical and experimental, should be conducted in order to understand and be able to predict their behavior.

## Figures and Tables

**Table 1:** Heat transfer enhancement with respect to water for functionalized graphene nanoplatelet aqueous nanofluids.

Nanofluid	Minimum	Average	Maximum
*φ_m_* = 0.25%	−1.7%	6.5%	18.6%
*φ_m_* = 0.50%	0.94%	15.0%	32.4%
*φ_m_* = 0.75%	−2.3%	7.0%	22.8%
*φ_m_* = 1.00%	−19.6%	–10.1%	4.0%

**Table 2:** Relative pressure drop in relation to pure water for graphene nanoplatelets water-based nanofluids.

Nanofluid	Minimum	Average	Maximum
*φ_m_* = 0.25%	0.92%	14.5%	55.4%
*φ_m_* = 0.50%	4.11%	17.1%	56.9%
*φ_m_* = 0.75%	13.0%	28.0%	73.4%

## Abbreviations

**Nomenclature**	
*a*	Annulus diameter ratio, dimensionless
*c_i_*	Correlation fitting constant, dimensionless
*c_p_*	Specific heat capacity, J·kg^−1^·K^−1^
*d*_1_	Inner diameter of the inner tube, m
*d*_2_	Outer diameter of the inner tube, m
*d*_3_	Inner diameter of the outer tube, m
*d_h_*	Hydraulic diameter, m
*f*	Darcy friction factor, dimensionless
*h*	Convection coefficient, W·m^−2^·K^−1^
*k*	Thermal conductivity, W·m^−1^·K^−1^
*LMTD*	Logarithmic mean temperature difference, °C
*L_h_*	Effective heating length, m
*L_p_*	Effective pressure drop length, m
Nu	Nusselt number, dimensionless
Pr	Prandtl number, dimensionless
$\dot{Q}$	Heat flow rate, W
*R*	Thermal resistance, K·W^−1^
Re	Reynolds number, dimensionless
*T*	Temperature, K, °C
*v*	Average velocity of the fluid, m·s^−1^
$\dot{V}$	Volume flow rate, m^3^·s^−1^
vol %	Percentual graphene nanoplatelets volume concentration, dimensionless
wt %	Percentual graphene nanoplatelets mass concentration, dimensionless
*Δp*	Pressure drop, Pa
*η*	Thermal performance factor, dimensionless
*ρ*	Density, kg·m^−3^
*φ_m_*	Graphene nanoplatelets mass concentration, dimensionless
*φ_v_*	Graphene nanoplatelets volume concentration, dimensionless
*ψ* | *ω*	Correction factors for the Nusselt number in the annulus, dimensionless
**Subscripts**	
*bf*	Base fluid
*hw*	Heating water
*in*	Inlet of the test section
*nf*	Nanofluid
*out*	Outlet of the test section
*ov*	Overall
*s*	Steel
*tf*	Tested fluid
*w*	Water
*wall*	Tube wall
